# Supplementary material for: PERK activation by SB202190 ameliorates amyloidogenesis via the TFEB-induced autophagy-lysosomal pathway
Source: Aging (Albany NY). 2022 Feb 15;14(3):1233–52. doi: 10.18632/aging.203899 (PMC8876914; doi:10.18632/aging.203899)
Supplement: Supplementary Figures [file aging-14-203899-s001.pdf]

SUPPLEMENTARY FIGURES

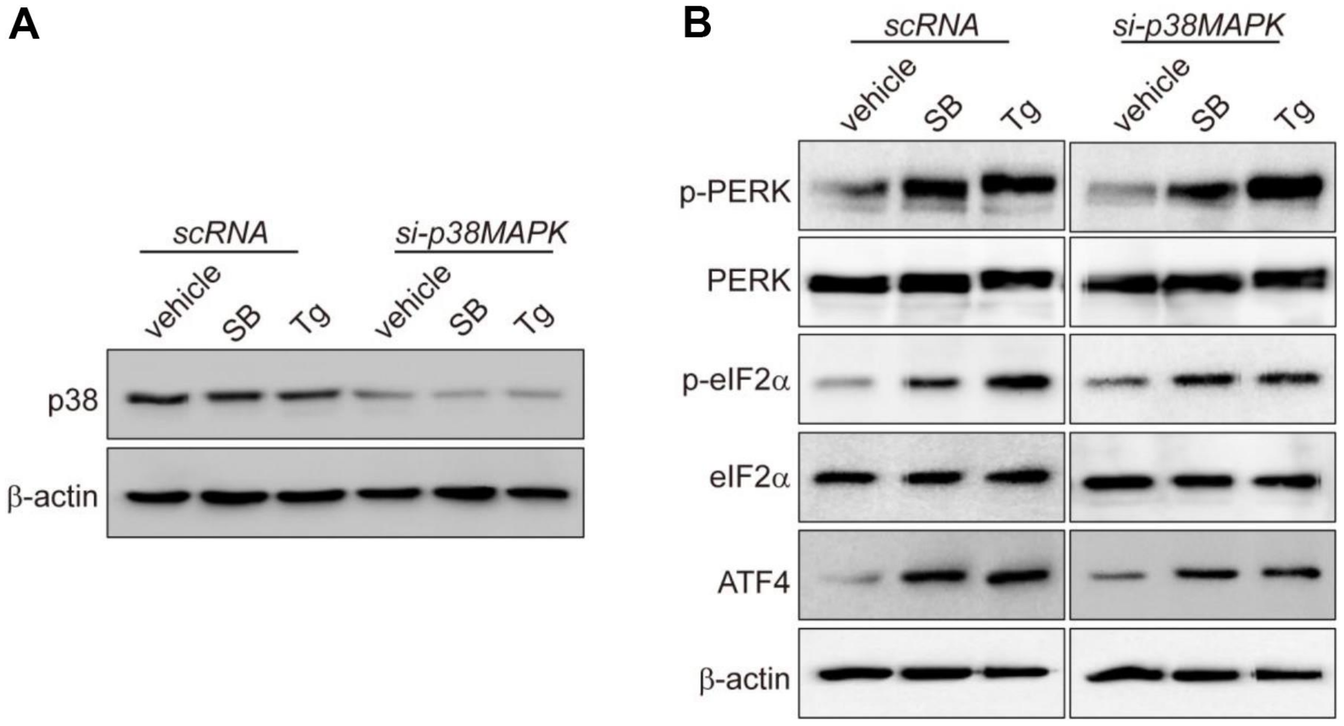

Supplementary Figure 1. PERK activation with SB202190 is independent of p38 MAPK-related to Figure 1. (**A**, **B**) SH-SY5Y cells were transfected with control siRNA (scRNA) or si-p38 MAPK for 48 h and then treated with 20 μM SB202190 for 6 h or 1 μM Tg for 30 min. Cell lysates were evaluated for p38 MAPK knockdown by western blot (**A**) and detected with antibodies against p-PERK, PERK, p-eIF2α, eIF2α, and ATF4 by western blotting (**B**).

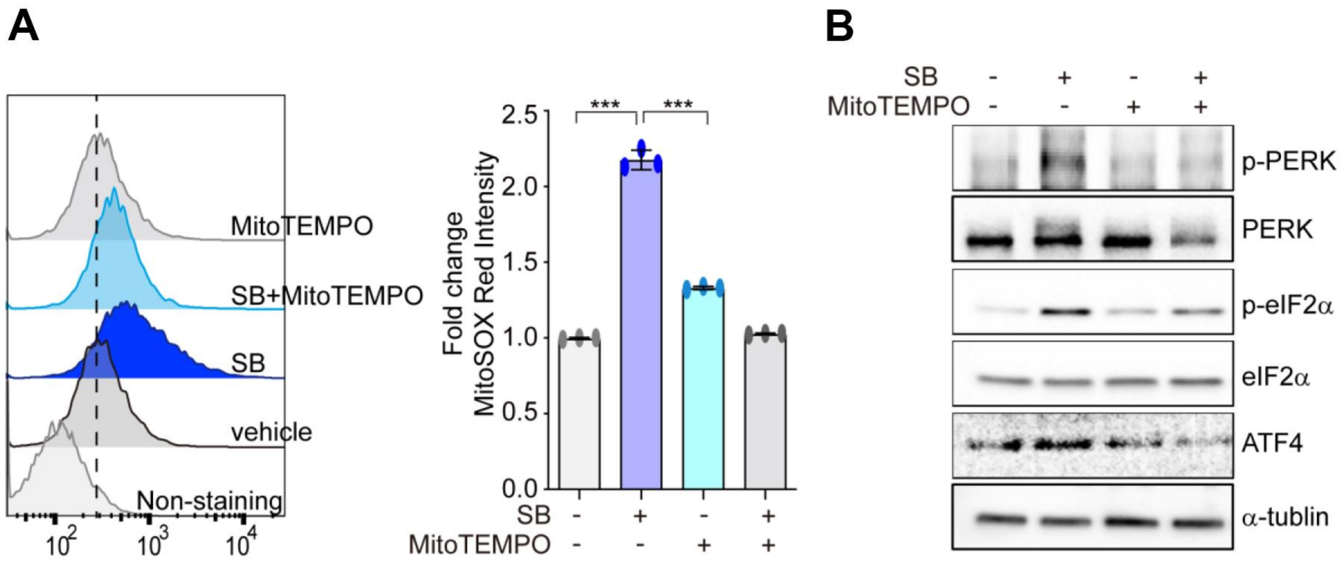

Supplementary Figure 2. PERK activation with SB202190 is induced by ROS production- related to Figure 2. (**A**, **B**) HEK293 cells were pretreated with MitoTEMPO (100 nM) for 1 h and then treated with 20 μM SB202190 for 3 h. (**A**) MitoSOX fluorescence was analyzed by flow cytometryd (left), and quantification of fluorescence was normalized (right). Data are mean ± SD (n=3), ***p<0.001. (**B**) The activation of PERK was analyzed by western blotting using the indicated antibodies.

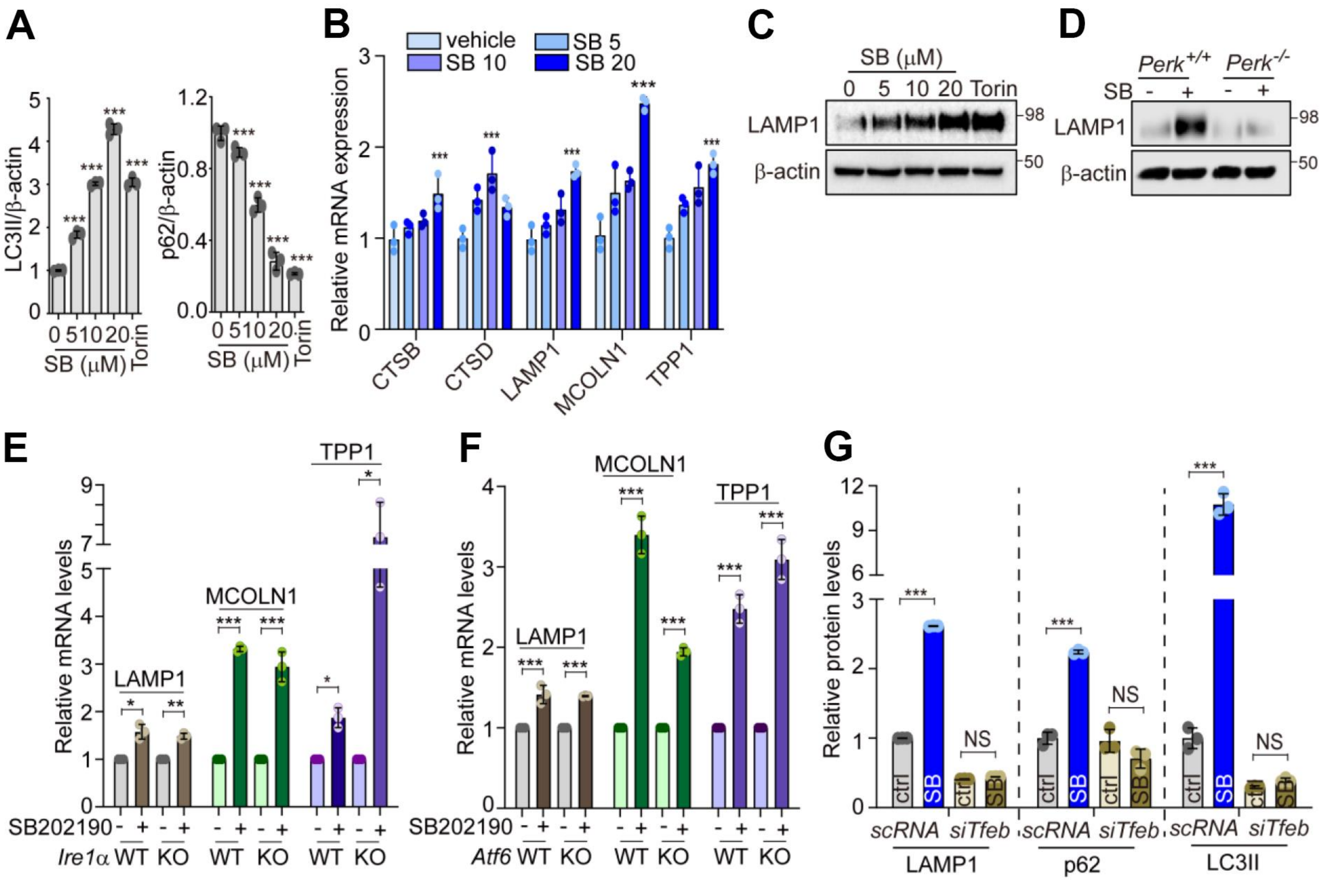

Supplementary Figure 3. PERK activation by SB202190 facilitates autophagy and lysosome biogenesis via TFEB activation related to Figure 5. (**A**) Western blotting shown in Figure 5A was analyzed to quantify the band densities. Data represent mean ± SD, ***p<0.001. (**B**) SH-SY5Y cells were treated with SB202190 (5, 10, and 20 µM) for 6 h, and then lysosomal genes were analyzed by qRT-PCR. Data represent mean ± SD; ***p<0.001. (**C**) SH-SY5Y cells were treated with SB202190 at the indicated concentrations for 6 h. Torin-1 (2 µM) was used as a positive control. The levels of LAMP1 and p62 expression were evaluated by western blotting. (**D**) *Perk*$^{+/+}$ and *Perk*$^{-/-}$ MEFs were treated with SB202190 for 6 h, and LAMP1 expression was analyzed by western blotting. (**E**) Western blotting shown in Figure 5E was analyzed to quantify the band densities. (**F**, **G**) Hepatocytes from *Ire1*α$^{+/+}$, *Ire1*α$^{-/-}$ (**F**), *Atf6*α$^{+/+}$, and *Atf6*α$^{-/-}$ (**G**) mice were treated with SB202190 (20 µM) for 6 h to assess the levels of LAMP1, MCOLN1, and TPP1 expression by qRT-PCR. Data represent mean ± SD; *p<0.05, **p<0.01, and ***p<0.001.

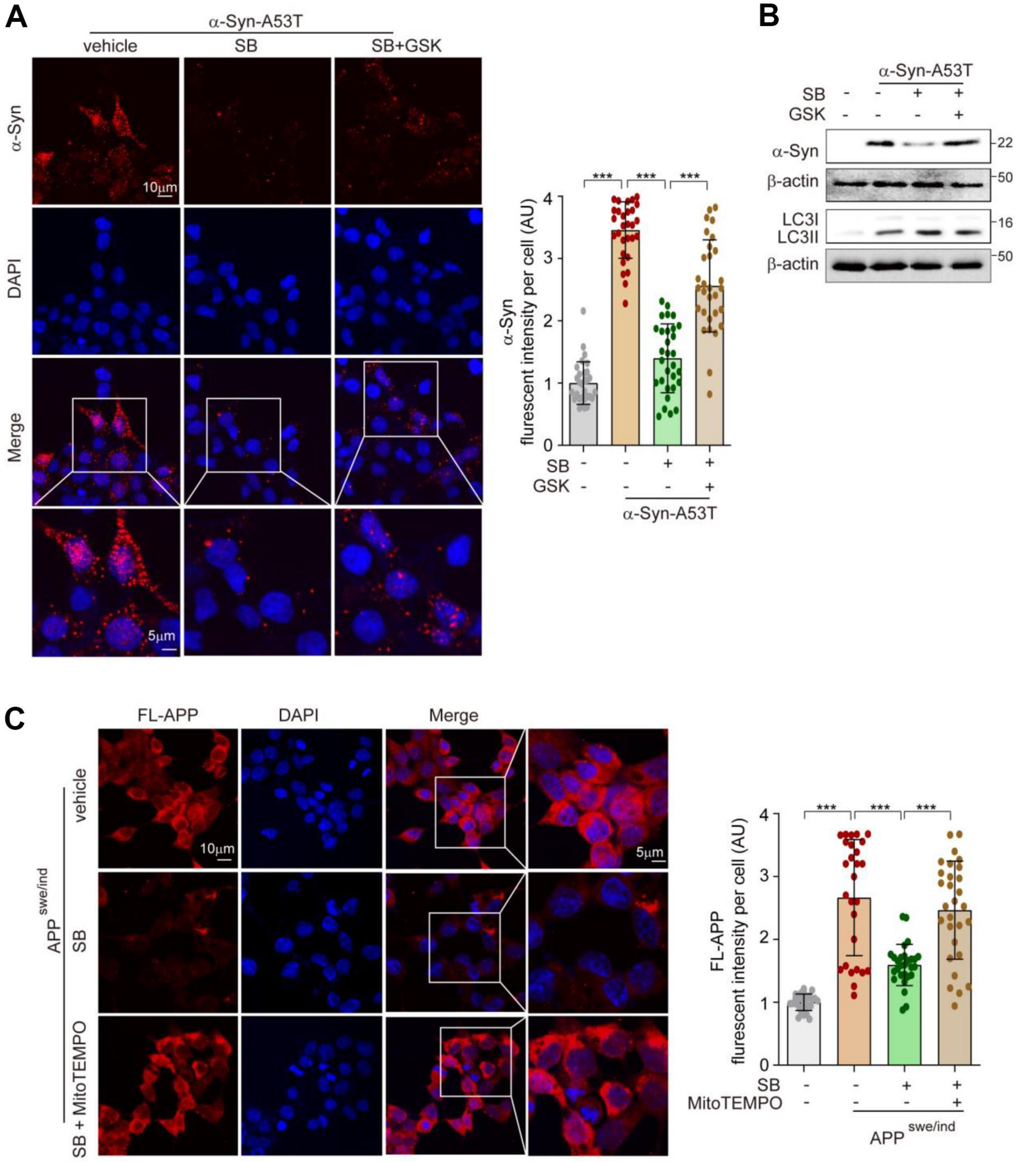

Supplementary Figure 4. PERK activation by SB202190 reduces the aggregation of APP and α-syn accumulation through TFEB-ALP activation in SH-SY5Y cells related to Figure 6. (**A**, **B**) SH-SY5Y cells were transiently transfected with α-Syn-A53T for 48 h. Cells were treated with GSK2606414 (1 μM) for 1 h before SB202190 (20 μM) treatment for 12 h. Representative image of α-Syn was detected by confocal microscopy (left) and quantification of α-Syn intensity (right). Data represent mean ± SD; ****p*<0.001. (**B**) The levels of α-Syn and LC3B-II conversion were analyzed by western blotting. (**C**) SH-SY5Y cells were transfected with pCAX-APP-Swe/Ind (APP$^{swe/ind}$) for 48 h. Cells were subsequently pretreated with MitoTEMPO (100 nM) for 1 h and then treated with SB202190 (20 μM) for 12 h. Representative image of FL-APP was analyzed by confocal microscopy (left) and quantification of FL-APP intensity (right). Data represent mean ± SD; ****p*<0.001.
